# Respiratory and hemodynamic effects of intravenous nalbuphin in anesthetized rats

**DOI:** 10.1186/2197-425X-3-S1-A640

**Published:** 2015-10-01

**Authors:** T Hauffe, P Jirkow, M Arras, D Müller, DR Spahn, D Bettex, A Rudiger

**Affiliations:** Institute of Anesthesiology, University Hospital Zurich, Zurich, Switzerland; Division of Surgical Research, University Hospital Zurich, Zurich, Switzerland; Institute for Clinical Chemistry, University Hospital Zurich, Zurich, Switzerland

## Introduction

Animal welfare mandates adequate analgesia in sepsis models (Lilley E, Shock 2015; 43: 304). However, it is unclear which regimen is best to achieve this without interfering excessively with the scientific experiments. Nalbuphin, a non-controlled kappa opioid receptor agonist, has a short half live and a wide therapeutic range and might therefore be a useful analgesic for research animals.

## Objectives

We test the efficacy and safety of intravenous nalbuphin in our rat model of fecal peritonitis. Here, we report its respiratory and hemodynamic effects in short-term experiments performed in non-septic rats during anaesthesia.

## Methods

In this randomized, placebo-controlled, blinded study, spontaneously breathing male Wistar rats were anesthetized with isoflurane 3% (air flow 400 ml/min, FiO2 21%). Nalbuphin (or placebo) was injected subcutaneously at a dose of 1 mg/kg. After local anaesthesia with lidocaine, the left carotid artery and the right jugular vein were surgically cannulated. The isoflurane concentration was reduced to 2%. Intravenous nalbuphin (or placebo) was administered at two different doses (1 and 5 mg/kg/h). Nalbuphin concentration was measured in heparinised plasma by HPLC. Results are given as mean (standard error of the mean), p-values were calculated by a t-test.

## Results

See table 1.

**Table 1:** Results.

	Placebo (n=6)	Nalbuphin 1 mg/kg/h (n=6)	p-value	Placebo (n=6)	Nalbuphin 5 mg/kg iv (n=6)	p-value
Nalbuphin concentration [mg/l]		22.9 (3.7)				
**Respiratory parameters**						
Respiration rate [1/min]	55 (3)	45 (2)	0.02	53 (4)	41 (2)	0.04
pO2 [kPa]	9.31 (0.41)	7.86 (0.98)	0.41	10.1 (0.66)	6.81 (0.36)	0.02
SaO2 [%]	92.7 (1.2)	83.9 (3.3)	0.09	93.6 (1.3)	78 (2.7)	0.02
pCO2 [kPa]	6.25 (0.31)	7.72 (0.52)	0.03	6.45 (0.53)	8.49 (0.39)	0.01
**Hemodynamic parameters**						
Heart rate (1/min)	409 (10)	336 (12)	0.00	413 (14)	332 (16)	0.01
Mean arterial pressure (mmHg)	95 (6)	78 (4)	0.04	92 (5)	70 (3)	0.01

## Conclusions

Our results demonstrate that high-dose nalbuphin depresses respiratory rate, heart rate and mean arterial pressure in anesthetised rats. In order to investigate if the continuous intravenous infusion of nalbuphin influences our long-term sepsis model, further placebo-controlled studies in awake septic and non-septic rats are ongoing.
